# Migraine: from pathophysiology to treatment

**DOI:** 10.1007/s00415-023-11706-1

**Published:** 2023-04-08

**Authors:** Francesca Puledda, Elisa Martins Silva, Kanokrat Suwanlaong, Peter J. Goadsby

**Affiliations:** 1 Headache Group, Wolfson CARD, Institute of Psychiatry, Psychology and Neuroscience, King’s College London, and National Institute for Health Research (NIHR) SLaM Clinical Research Facility at King’s, Wellcome Foundation Building, King’s College Hospital, London, SE5 9PJ UK; 2 Department of Neurology, Hospital Garcia de Orta, Almada, Portugal; 3 Division of Neurology, Department of Medicine, Songkhla Medical Education Center, Songkhla, Thailand; 4 Department of Neurology, University of California, Los Angeles, Los Angeles, CA USA

**Keywords:** Migraine, Pathophysiology, Treatment, CGRP, Neuromodulation

## Abstract

Migraine is an extremely disabling, common neurological disorder characterized by a complex neurobiology, involving a series of central and peripheral nervous system areas and networks. A growing increase in the understanding of migraine pathophysiology in recent years has facilitated translation of that knowledge into novel treatments, which are currently becoming available to patients in many parts of the world and are substantially changing the clinical approach to the disease. In the first part of this review, we will provide an up to date overview of migraine pathophysiology by analyzing the anatomy and function of the main regions involved in the disease, focusing on how these give rise to the plethora of symptoms characterizing the attacks and overall disease. The second part of the paper will discuss the novel therapeutic agents that have emerged for the treatment of migraine, including molecules targeting calcitonin gene-related peptide (gepants and monoclonal antibodies), serotonin 5-HT_1F_ receptor agonists (ditans) and non-invasive neuromodulation, as well as providing a brief overview of new evidence for classic migraine treatments.

## Introduction

Migraine is currently listed as the sixth most disabling disorder globally, with the highest ranking among all neurological disorders [1]. The biology of migraine is complex, multifactorial and still, for certain aspects, unsolved. The underlying feature seems to be a, probably complex, genetic predisposition combined with behavioral and environmental conditions that causes an alteration of sensory brain processing, resulting in increased sensory susceptibility. This in turn results in otherwise normal sensory inputs being perceived as bothersome in migraineurs [2].

Over the years, our knowledge around migraine has improved considerably, largely thanks to basic science and imaging studies allowing us to better understand the complex models that are needed to explain the plethora of migraine symptoms. In fact, pain, the cardinal symptom of the disorder, is not necessarily the most bothersome for all patients at all times [3, 4]. Migraine is characterized by a succession of key phases that often overlap: the premonitory (prodromal), aura, pain and postdromal phases [5, 6]. Better recognition of these events has allowed us to conceptualize migraine as a network disorder involving multiple cortical, subcortical and brainstem regions, generating a wide constellation of signs and symptoms [7]. These areas, which we will analyze in detail in the following sections, have altered function and structure in individuals with migraine and in animal models of the disease.

The current review follows a translational and anatomical approach, beginning with an outline of the mechanisms and regions that are known to be a part of migraine biology, before moving on to current acute and preventive treatments, providing updated references and insights with respect to our previous review of 5 years ago [8].

## Migraine: functional anatomy and pathophysiology

### The trigeminovascular system and brainstem nuclei

The trigeminovascular system consists of peripheral axons from the trigeminal ganglion that innervate the meninges and intracranial blood vessels peripherally, and converge centrally in the trigeminocervical complex (TCC), composed of the spinal trigeminal nucleus caudalis and upper cervical spinal cord [9, 10]. Second-order neurons ascend from the TCC to thalamocortical neurons and further project to key brain nuclei in the diencephalon and brainstem, such as the locus coeruleus (LC), periaqueductal gray (PAG) and hypothalamus [11, 12]. Activation of the trigeminovascular pain pathways is thought to mediate part of the qualities of migraine pain by release of neuropeptides, such as calcitonin gene-related peptide (CGRP) and pituitary adenylate cyclase activating polypeptide (PACAP), at the level of the dura mater [13–15]. CGRP is widely expressed in both peripheral and central neurons and has potent dilatator qualities. It also shows a regulatory action on second- and third-order neurons, which seems to underlie its modulatory role in central pain mechanisms. CGRP elevation in migraineurs has been linked to a decrease in descending inhibitory mechanisms, which in turn might lead to migraine susceptibility through sensitization of multiple central neuronal circuits [13].

Neurogenic inflammation in the periphery was initially proposed to be the generator of migraine pain [16], although this role has been revisited largely due to the fact that blockers of plasma protein extravasation have failed to treat migraine in clinical trials [17, 18]. While trigeminal activation with associated neurogenic inflammation continues to be discussed [19], direct evidence for a dural inflammatory component in migraine is lacking. As described above, migraine is associated with a spectrum of sensory dysfunctions with cycling behavior, during which the headache phase represents the plateau of trigeminal nociceptive activation [20]. One hypothesis is that peripheral trigeminovascular neurons are sensitized, and thereafter sensitize second-order neurons in the trigeminal nucleus caudalis and upper cervical spinal cord, and project rostrally to thalamic nuclei and key medullary, brainstem and diencephalic regions [21]. Studies have reliably provided evidence for early brainstem involvement in the nociceptive migraine phase; however, it is becoming clearer that the initiation of a migraine attack is linked to intrinsic brain dysfunction in more central areas such as the hypothalamus, and possibly to external factors as well [22]. Whether the central dysfunction, very clearly demonstrated in the premonitory phase, facilitates central sensitization remains an intriguing area of study.

The role for brainstem regions, such as the PAG and the dorsolateral pons in migraine is well established thanks to observational [23] and neuroimaging studies [24–26], as well as animal models of migraine showing that the brainstem acts as a driver of changes in cortical activity during migraine [27–30]. Indeed, nuclei such as the LC, rostral ventral medulla, superior salivatory and cuneiform nucleus are key in modulating trigeminovascular pain transmission and autonomic responses in migraine and represent a site of action for triptans [31, 32], ergot derivatives [33, 34] and the novel CGRP receptor antagonists [35, 36]. Further, direct and indirect trigeminal activation of the parabrachial nucleus may explain why head and facial pain is so intense when compared with noncephalic pain, whereas upward trigemino–parabrachial–limbic connections, particularly to the amygdala, can explain affective-motivational aspects of migraine and even appetite and taste abnormalities [12]. Linking the premonitory phase and the onset of pain, neurons in the ventral tegmental parabrachial pigmented (VTA^PBP^) nucleus can modulate trigeminocervical nociceptive traffic in rat. These effects can be seen with glutamate, naratriptan (5HT_1B/1D_ receptor agonist), PACAP and dopamine D_2/3_ mediation [37]. Given the role of the VTA^PBP^ in hedonic feeding, and the influence of glucose on trigeminocervical nociceptive transmission [38], the data suggest plausible pathways to explain the much celebrated, yet seldom clinically useful, issues of food triggers [39]. One might re-think some triggers in terms of behaviors arising from central nervous system activation in the premonitory phase [40], with very different conclusions concerning cause and effect. Finally, central sensitization of the trigeminovascular system, especially the trigeminal nucleus caudalis, plays an important role in the development of chronic migraine, possibly influenced by cytokines release and increased astrocytic activation [41, 42]. Interestingly, as shown by neurophysiological [43] and neuroimaging studies [44], brainstem activations seems most prominent in the 24 h preceding the headache onset, and declines during the attack.

### The hypothalamus

The theories on migraine as a cyclic sensory threshold disorder have highlighted the importance of the hypothalamus as a central facilitator of pain, and also of the constellation of premonitory symptoms such as yawning, thirst and polyuria [45], which can precede and continue into the pain phase. Functional neuroimaging performed during spontaneous and nitroglycerin-triggered attacks has consolidated the role of the hypothalamus in migraine initiation [46]. Altered hypothalamic-brainstem connectivity with the spinal trigeminal nuclei and the dorsal rostral pons has been shown in the premonitory phase of migraine [44] for up to 48 h preceding pain onset [47]. Positron emission tomography has also previously revealed hypothalamic activation during both spontaneous migraine headache [48] and the premonitory phase [49]. Recent functional imaging studies have even shown that changes in hypothalamic connectivity with the spinal trigeminal nucleus and cortical regions are associated with the development of chronic migraine [50, 51].

The mechanism(s) by which the hypothalamus can become ‘overactive’ in migraine, leading to sensitization of trigeminal nociceptors is still unclear. Anatomically, the hypothalamus has direct and indirect connections to the thalamus [52], the trigeminovascular system [53] and to sympathetic and parasympathetic brainstem neurons [54], influencing nociceptive and autonomic regulation in migraine. Stress, which is said to be a common trigger of migraine, can activate the kappa opioid receptor on tuberoinfundibular dopaminergic neurons and lead to an increase in circulating prolactin causing sensitization of trigeminal afferents, particularly in females [55]. Further, the hypothalamus has chemosensitive neurons that can detect metabolic changes in the brain and periphery. Exogenous stimuli causing a change in homeostasis and this intrinsic biorhythm could thus possibly ‘tip’ the brain towards a migraine attack via activation of the hypothalamus [56].

### The thalamus

The thalamus has a critical role in sensory processing, receiving inputs from the extracranial skin and dura mater from second-order trigeminovascular neurons, and projecting to cortical regions involved in autonomic, affective, and cognitive functions—all of which explains in part the complexity of migraine features [57]. Thalamocortical synchronization is affected by a network of neurotransmitters and neuropeptides in the brainstem (glutamate, serotonin, and noradrenaline), reticular thalamic regions (γ-aminobutyric acid—GABA) and hypothalamic nuclei (dopamine, histamine, orexin, and melanin-containing hormone [58]). There is abundant clinical and preclinical evidence showing that the thalamus is crucial for the development of central sensitization, photophobia and allodynia in migraine [59–64].

Structural neuroimaging studies have shown differences in volume of thalamic nuclei with microstructural abnormalities [65–67]. However, such changes were not seen in a recent large study involving female patients with aura [68]. Functional MRI studies have also shown important changes in the thalamus, both within and outside of attacks. In migraine without aura, connectivity between the thalamus and pain modulating areas seems to be affected during the ictal phase [69]. Abnormal low-frequency oscillations in dynamic thalamocortical networks are implicated in the interictal phase [70], with changes in pulvinar activity allowing differentiation between migraineurs and controls [71]. Another recent study showed that both episodic and chronic migraine patients have greater activation of ascending trigeminal somatosensory pathways and lower activation of top-down pain modulatory circuits. This could indicate interictal dysfunction of the descending pain modulatory system and amplification of nociceptive processing in migraineurs, mediated by the thalamus and possibly contributing to central sensitization [72].

The processing of trigeminovascular nociceptive information in the thalamus can represent a target for management, and indeed several migraine treatments including triptans [31, 73], preventives [74–77] and non-invasive neuromodulation have been shown to modulate thalamocortical activity [78, 79].

### The cortex

The role of the cerebral cortex in migraine was initially linked to the aura phenomenon and its peculiar symptoms [80, 81]. Aura is thought to be generated by cortical spreading depression (CSD) [82], which has been indirectly evidenced in humans through functional neuroimaging [83]. Although CSD can activate the trigeminovascular system in animals [84, 85] possibly through CGRP-mediated mechanisms [86], it is unlikely to contribute to the headache and possibly constitutes an epiphenomenon of migraine [87, 88].

Regardless of CSD, the cortex has been increasingly implicated in migraine genesis, and in fact many changes in the structure and function of key cortical areas associated with pain processing have been reported in patients, both in the ictal and interictal period [89]. During the headache phase, cortical networks including the salience, sensorimotor, default mode, executive and attentional networks, show functional changes; this reflects the cognitive, painful and emotional symptoms of migraine [90]. Studies in patents with aura have consistently shown differences in brain structure [91, 92], functional connectivity [92], cortical excitability [93–95] and pain modulation in the visual pathways [96]. Occipital cortex involvement in particular can explain the plethora of visual symptoms associated with migraine, from light sensitivity to visual aura and visual snow [97]. Menstrual migraine has recently been linked to structural and functional connectivity changes in the right anterior cingulum [98] an area involved in the cognitive processing of pain and previously associated with migraine biology [99]. However, evidence from neuroimaging studies has been inconclusive at times [100], with meta-regression analyses failing to pinpoint alterations that are specific to migraine [101], showing that further research on the topic is needed.

Of note, the association of white matter hyperintensity (WMH) in migraine has long been debated [102], particularly in migraine with aura [103, 104]. Recently, an association was identified between the presence of juxtacortical WMHs within the frontal lobe with patient age and duration of disease [105]. WMHs have also been associated with nausea, vomiting, dizziness and pain intensity during attacks, [106].

Dysfunctional cortical mechanisms and in particular thalamocortical dysrhythmia have also been implicated in the mechanism underlying the lack of habituation typical of migraine [107]; in this, repeated sensory stimuli cause an incremental, instead of a reduction, increase in the amplitudes of sensory responses [108, 109]. Lack of habituation, measured for different sensory modalities, usually occurs during the pain-free period and reverts during the ictal phase or when attacks become more frequent [110].

Finally, large genome-wide association studies (GWAS) have identified susceptibility gene variants in migraine patients, mostly involved in glutamatergic neurotransmission, which could lead to abnormal cortical excitability and altered plasticity [111], as evidenced by numerous magnetic resonance spectroscopy studies performed over the years [112]. Readers interested in the genetics are referred to this recent article [113]. Given the complexity that has emerged from GWAS work, in which each change has such a modest effect on the overall phenotype, one might reflect on whether clinically useful genetic changes will emerge in the near term that will have an impact on management and treatment.

## Novel therapies in migraine

The last few years have represented an exciting and promising time in the field of migraine, thanks to the introduction of several new medications in clinical practice, and with other therapeutic targets, such as glutamate, amylin, adrenomedullin, orexins and pituitary adenylate cyclase activating polypeptide, currently all in the therapeutic pipeline [114, 115]. The new novel treatments have rapidly changed the paradigm of migraine management, particularly challenging the dichotomous division between acute and preventive medication, which we will however, follow in this review for simplicity. Further, patient-reported outcomes such as interictal burden and time lost due to an attack are becoming more relevant in the consideration of efficacy and tolerability of these drugs [116, 117], allowing for significant advances in the management of this condition [118].

### Acute treatments

Therapy for migraine attacks includes non-steroidal anti-inflammatory drugs (NSAIDs), combination analgesics, ergotamine preparations and migraine-specific medications. The latter class, which until a few years ago meant triptans, has recently grown to include ditans, serotonin 5HT_1F_ receptor agonists, and gepants, CGRP receptor antagonists. Triptans are full agonists of presynaptic serotonin receptors 5-HT_1B_ and 5-HT_1D_ [119], which inhibit CGRP release [120]. The class includes seven options in different formulations [121], which can be switched to find the optimal combination for efficacy and tolerability in the individual patient and which can be combined with NSAIDs to prolong therapeutic effect and limit rebounds [122, 123]. There are gender-related differences in triptan tolerability, as women seem to present higher adverse event frequency and headache recurrence rates with these drugs [124].

Non-responsiveness to triptans may be categorized into refractory: failure of three triptans, one of which should be subcutaneous sumatriptan; and resistant: failure of at least two triptans [125]. Non-responsiveness can have a significant impact on health-related quality of life and work productivity [126] and has been linked by recent neuroimaging data to changes in hippocampal volume [127]. Importantly, even if clinical practice has not demonstrated strong drug-related cardiovascular risk [128], triptans are still contraindicated in at-risk patients due to their vasoconstrictive qualities [129]. The new classes of ditans and gepants do not present this disadvantage.

#### Ditans

Lasmiditan is the only ditan currently available; it is a potent and selective 5-HT_1F_ receptor agonist [130], acting in migraine by blocking activation of neurons in the trigeminal nucleus caudalis [131] without affecting the vasculature [130]. Lasmiditan has now been studied in two-phase two studies [132, 133] and three large phase three randomized controlled trials [134–136] and shown to have better efficacy compared to placebo on rates of 2-h pain freedom and freedom from most bothersome symptoms, particularly with the 100 and 200 mg doses. Pooled data from the phase 3 studies showed no cardiovascular safety concerns, and in fact, these included patients with coronary artery disease, complicated cardiac arrhythmias and/or hypertension [137]. Across these studies, neurological side effects—particularly dizziness, nausea and somnolence—were common, but mostly mild to moderate and self-limiting [138].

#### Gepants

Gepants are small-molecule CGRP receptor antagonists developed for use in the acute treatment of migraine. Six gepants were initially developed for acute use in migraine, with two being discontinued due to liver toxicity [139, 140], one due to lack of oral availability [141] and one for commercial reasons [142]. Ubrogepant and rimegepant represent a new generation of oral gepants that have received FDA approval for acute migraine therapy [143, 144] following phase 3 studies: Achieve I [145] and II [146] for ubrogepant, and Study 301 [147], 302 [148] and 303 [149] for rimegepant. Ubrogepant has been approved at 50 and 100 mg doses and Rimegepant is available in an orodispersible (lyophilized) form at a dose of 75 mg. Preliminary evidence also shows effectiveness of the zavegepant nasal spray, a non-oral gepant [150]. Importantly, gepants do not seem to cause medication overuse headache, making them a useful option when managing this complication [151] and can be taken in multiple doses during the attack with good rates of success [152]. The low side effect profile of gepants is appealing; however, caution in the early days of real-world use is merited [153].

Metabotropic and ionotropic glutamate receptors may become important targets in the future acute therapy of migraine, although adverse event issues need to be overcome. Experimental and clinical studies have shown an effect of NMDA, AMPA, iGluR5 and mGluR5 receptor antagonists in migraine, although their efficacy was lower than that of sumatriptan and visual side effects were observed [154–156]. Blockers of the metabotropic glutamate receptor 5 in particular, or *glurants*, have a strong clinical potential for becoming a candidate drug class for migraine, if the relevant issues of hepatoxicity and transient dizziness can be resolved [157]. The NMDA receptor is also relevant for migraine with aura, as evidenced by a small RCT showing the efficacy of ketamine on reducing the severity of auras [158].

### Preventive treatments

Preventive therapy is recommended in patients who are affected by migraine on at least 2 days per month, when there is medication overuse and/or when quality of life is impaired [159]. The application of this guidance will be governed by clinical judgment in the individual case. Classic prevention includes different drug categories, such as β blockers, anticonvulsants, tricyclic antidepressants and calcium channel modulators, which, however, often lead to tolerability issues and poor compliance [160]. In recent years, monoclonal antibodies (mABs) against the CGRP peptide (galcanezumab, fremanezumab, eptinezumab) or its canonical receptor (erenunmab) have been widely introduced in clinical practice and treatment guidelines [161]. These treatments have persistently confirmed their efficacy in phase 3 trials [162–174], with convenient dosing, faster onset of efficacy and mild to moderate adverse events [175, 176]. Further, real-world studies have shown improvement with mABs and worsening of migraine frequency following discontinuation, with most patients resuming treatment as soon as possible following breaks due to regulatory restrictions [177–180]. The European Headache Federation currently recommends CGRP mABs as a first-line option for migraine prevention, with treatment to be continued as long as needed [181], although in most jurisdictions, this is not possible to operationalize easily.

Two gepants, atogepant and rimegepant, have recently been introduced into the market after proving effective and well tolerated for the preventive treatment of migraine [182–184]. Both have a similar short half-life of around 11 h [185, 186], which facilitates their preventive indication. They bring the advantage of fewer adverse events and increased safety, particularly in women who have unplanned pregnancies given the difference in half-life compared to monoclonal antibodies [151]. Atogepant was directly designed as a preventive agent, and has been FDA-approved for episodic migraine [187]. The most common side effects in the phase 2b and 3 studies were constipation and nausea, each at 10% at the 60 mg daily dose. The currently available data suggests safety on cardiac repolarization even with supratherapeutic doses and, in contrast with the first gepants, no elevation of serum alanine aminotransferase [188, 189]. A further study for the preventive treatment in chronic migraine has been reported in abstract form [190]. It is being studied in combination with onabotulinumtoxinA (NCT05216263) and for long-term safety and tolerability in another trial (NCT04686136).

Rimegepant, initially trialed for acute use, can prevent episodic migraine in adults when taken every other day [184] with the additional benefit of it being used concomitantly during a migraine attack. It is well tolerated, with nausea occurring in 2% of cases and this is the most common side effect.

New evidence on efficacy and tolerability has also emerged for well-known migraine preventives. A meta-analysis has documented the efficacy in chronic migraine of OnabotulinumtoxinA, which allows for a reduction of over 50% in migraine days after 24 weeks of treatment [191]. Several open-label studies have also shown a benefit of combining onabotulinumtoxinA with mAbs to CGRP for CM [192–194]. A recent head-to-head trial for chronic migraine showed non-inferiority between propranolol and topiramate, with no significant difference in adverse events incidence between the two [195]. Another prospective randomized trial in chronic migraine compared flunarizine 10 mg with topiramate 50 mg daily, showing both drugs had a similar safety profile, with flunarizine being overall more effective [196]. Recent retrospective studies have confirmed the usefulness of candesartan as a first-line migraine preventive, even in patients who failed numerous previous drugs [197, 198]. Finally, meta-analyses have been conducted on melatonin [199, 200] and memantine [201], both showing favorable side effect profiles and good efficacy in migraine.

### Neuromodulation

Non-invasive neuromodulation is an evolving field and is of particular clinical interest for migraine management as it offers the option of being used both as an acute and preventive treatment. It also presents near to no systemic side effects and can thus be offered to patients that present tolerability issues or who need to avoid medication interaction [202].

The devices used in migraine target the nervous system through a transcutaneous approach, either centrally (single-pulse transcranial magnetic stimulation, or sTMS) or in the periphery (non-invasive vagus nerve stimulation or nVNS, supraorbital nerve stimulation or SNS and transcranial direct current stimulation or tDCS).

A handheld sTMS device is now approved in the USA and Europe for the acute and preventive treatment of migraine, following positive results as an acute migraine treatment in a RCT involving 164 migraineurs with aura [203] and subsequent post-marketing survey [204] and open-label study [205] demonstrating an effect on headache day reduction and 50% responder rate. The efficacy of sTMS in migraine prevention has also been shown in difficult-to-treat patients [206].

External trigeminal (supraorbital) nerve stimulation has also shown promise with supporting evidence for the treatment of migraine, with one RCT showing higher efficacy and tolerability than sham in 109 patients after 1 h of acute treatment [207]. For prevention, its effect seems greater in episodic migraine [208] than in refractory [209] or chronic migraine patients [210].

Regarding non-invasive vagus nerve stimulation (nVNS), it has shown evidence of efficacy in a RCT for the acute treatment of migraine [211], but not for prevention [212–214]. From a mechanistic perspective, this approach at the bench can suppress cortical spreading depression [215] and inhibit trigeminocervical neurons responding to durovascular nociceptive activation [216].

Another approach has focused on the application of repeated cathodal or anodal transcranial direct current stimulation over the cortex, although data on its therapeutic effect in migraineurs has been conflicting [217, 218]. This may be due to methodological differences regarding the techniques, the targeted brain regions and stimulation types [219], warranting further investigation.

Novel options for neuromodulation include the remote noncephalic electrical neurostimulation of the upper arm skin. The device works non-invasively through conditioned pain modulation and [220] has been evaluated in a RCT involving 253 patients. Participants reported clinically meaningful relief from migraine pain and pain freedom after 2 h of treatment compared to sham, with a low incidence of device-related adverse events [221]. A recent open-label study showed preliminary evidence supporting its use in chronic migraine [222]. Finally, repetitive peripheral magnetic stimulation (rPMS) targeting the muscles in the neck and shoulder muscles has shown efficacy in the prevention of episodic migraine, particularly in patients with a high level of muscular involvement [223, 224].

Although these techniques are promising for the management of a disabling condition with often little treatment options, further evidence is needed to evaluate the scope of their effect in migraine, including novel mechanisms and targets [225, 226].

## Conclusions

The last 2 decades have been an incredibly exciting period for clinicians and researchers interested in migraine, as they have seen a rapid increase in studies that have led to a greater knowledge and understanding of the neurobiology of the disorder. From suffering with a condition that was often overlooked and under-managed, migraineurs are now being offered novel treatments that are more and more tailored to their needs, and that are fundamentally re-shaping our approach to the disease.

More research and progress is needed and is expected in the coming years, and hopefully this will continue to raise awareness around a complex phenomenon affecting millions of people all over the world.
